# Effectiveness of the COVID-19 Community Vulnerability Index in explaining COVID-19 deaths

**DOI:** 10.3389/fpubh.2022.953198

**Published:** 2022-09-23

**Authors:** Jinghua An, Shelley Hoover, Sreenivas Konda, Sage J. Kim

**Affiliations:** ^1^Department of Biobehavioral Nursing Science, College of Nursing, University of Illinois at Chicago, Chicago, IL, United States; ^2^School of Public Health, University of Illinois at Chicago, Chicago, IL, United States; ^3^Division of Epidemiology and Biostatistics, School of Public Health, University of Illinois at Chicago, Chicago, IL, United States; ^4^Division of Health Policy and Administration, School of Public Health, University of Illinois at Chicago, Chicago, IL, United States

**Keywords:** COVID-19 mortality, social vulnerability index, community vulnerability, negative binomial regression, random forest

## Abstract

**Objectives:**

To explore the effectiveness of a COVID-19 specific social vulnerability index, we examined the relative importance of four COVID-19 specific themes and three general themes of the COVID-19 Community Vulnerability Index (CCVI) in explaining COVID-19 mortality rates in Cook County, Illinois.

**Methods:**

We counted COVID-19 death records from the Cook County Medical Examiner's Office, geocoded incident addresses by census tracts, and appended census tracts' CCVI scores. Negative binomial regression and Random Forest were used to examine the relative importance of CCVI themes in explaining COVID-19 mortality rates.

**Results:**

COVID-19 specific Themes 6 (High risk environments) and 4 (Epidemiological factors) were the most important in explaining COVID-19 mortality (incidence rate ratio (IRR) = 6.80 and 6.44, respectively), followed by a general Theme 2 (Minority status & language, IRR = 3.26).

**Conclusion:**

The addition of disaster-specific indicators may improve the accuracy of social vulnerability indices. However, variance for Theme 6 was entirely from the long-term care resident indicator, as the other two indicators were constant at the census tract level. Thus, CCVI should be further refined to improve its effectiveness in identifying vulnerable communities. Also, building a more robust local data infrastructure is critical to understanding the vulnerabilities of local places.

## Introduction

The concept of social vulnerability has been widely used to understand differential exposure and outcomes of COVID-19 since the beginning of the pandemic (1, 2). However, accurately quantifying social vulnerability remains a challenge (3). The CDC's Social Vulnerability Index (SVI) is commonly used to identify vulnerable communities quantitatively (4). The SVI provides a composite score that incorporates 15 social indicators aggregated at a geographic scale (4). The four SVI themes were designed to quantify social conditions contributing to the effects of disasters; the 15 indicators include measures of socioeconomic status, household composition, racial/ethnic composition, and transportation conditions. Because many of these factors interact, the SVI themes represent groups of related indicators (4–6). A key strength of the SVI is its ability to take into account social conditions that determine communities' exposure to a disaster and capacity to respond and recover from a disaster (7).

Because the SVI measures a general level of vulnerability, incorporating disaster-specific indicators may improve its accuracy in predicting and explaining a particular disaster risk (3). One example is the Surgo Ventures' COVID-19 Community Vulnerability Index (CCVI), which modifies CDC's SVI by adding several COVID-19 specific indicators (6). The CCVI consists of seven themes: three (Themes 1–3) rearranged from the CDC's SVI and an additional four (Themes 4–7) that are COVID-19 specific. The CCVI aggregates 40 indicators into seven themes, which were then weighted equally to calculate a composite score at the census tract, county, or state levels (5, 6, 8). The Supplementary Table summaries CCVI themes and their indicators. Because theme scores are generated using the sum of percent ranks of indicators, these scores range from zero to one, with higher values indicating more vulnerability (6).

While the CCVI attempts to improve identifying areas with greater vulnerability to the current pandemic, to the best of our knowledge, there have been no efforts to examine the relative importance of the CCVI themes. In recent literature, the CCVI has been compared against other vulnerability indices that were developed for measuring community risks for poor COVID-19 outcomes, including the COVID Local Risk Index (9) and Pandemic Vulnerability Index (10). Validating the CCVI is helpful to ascertain the extent of practical applications of the index and identify areas for future refinement (11). A common validation approach is to compare the overall index score against the outcomes of a disaster that has occurred (12). One shortcoming of this approach is that it does not examine the relative contribution of each theme (domain) to the disaster outcome (12). With regard to the CCVI, this validation approach would not allow us to understand how the additional disaster-specific indicators expand our knowledge around COVID-19 outcomes and social vulnerability to them.

In this study, we employed a new approach to validating indices by exploring the relative contribution of multiple themes of an index, in this case, CCVI. In particular, we paid attention to the usefulness of the four disaster-specific themes in explaining COVID-19 mortality rates.

Cook County, the second largest county in the U.S. with a population of over 5.1 million, had the highest COVID-19 cases and deaths in Illinois (13). Among the 3,142 U.S. counties, Cook County ranks 2,600th (14); it was in the third quartile of the COVID-19 vulnerability, based on the Surgo Venture's CCVI. Nearly 12% of Cook County residents live below the federal poverty line. Whites account for 41.5% of the County population, followed by 26.0 Latinx and 23.7% Black (15). Racial/ethnic disparities in COVID-19 infection and deaths have been well documented in Cook County, particularly in the city of Chicago (1, 16, 17). Since the first COVID-19 death in Cook County on March 17, 2020, a total of 8,356 COVID-19 deaths were recorded in 2020 and an additional 4,587 in 2021 (18). Our primary aim of this paper was to understand the relative importance of census tract characteristics contributing to local vulnerability to the pandemic in Cook County.

## Methods

### Dataset and variables

The COVID-19 death records from March 17, 2020, to March 16, 2021, were retrieved from the Cook County Medical Examiner's Office (MEO) (18). The Medical Examiner Case Archive contains information about deaths occurred in Cook County that were under the MEO's jurisdiction. The MEO investigates human deaths that fall within certain categories, such as diseases constituting a threat to public health (19). The selected one-year period roughly reflects COVID-19 deaths in Cook County before the vaccines became widely available to the public. Death records were removed if their address was missing or incomplete. A total of 8,752 COVID-19 deaths in Cook County were included.

Addresses of the COVID-19 death records were geocoded using a geographic information system, QGIS v.3.10.7. Using the U.S. census tract numbers, we merged the seven CCVI themes with the cumulative death counts at the census tract level. The CCVI scores were obtained from Surgo Venture's publicly available CCVI dataset (14), in which CCVI scores were missing for 35 census tracts. Thus, 1,284 out of 1,319 census tracts were included in the analyses.

### Statistical analysis

All analysis was conducted at the census tract level. The COVID-19 mortality rate (number of COVID-19 deaths per 100,000 residents) was computed for each census tract. The distribution of the seven CCVI themes for census tracts of Cook County was summarized. Spearman's rank correlation was used to examine the monotonic associations among the seven themes. To examine the effectiveness of the CCVI in explaining the COVID-19 mortality rates, negative binomial regression and Random Forest methods were selected from several analysis methods (20, 21). Akaike Information Criterion (AIC), a measure of model fit, was used for model selection. We defined the predictive accuracy of a model as its ability to predict the outcome accurately. Predictive accuracy in this paper was measured by the square of Spearman's rank correlation between true mortalities and model predictions.

The negative binomial regression provided the lowest AIC among other regression models (Multiple linear, Poisson, Quasi Poisson, and zero-inflated Poisson regression) to predict the COVID-19 mortality rates with overdispersion. To validate the significance and importance of each CCVI theme and to account for heterogeneity in the census tracts, we selected Random Forest after comparing its predictive accuracy with that of other machine learning methods, including Decision Tree, Bagging, and Neural Networks. The statistical analysis was performed using R 4.1.2.

## Results

Overall, considerable overdispersion was observed in the COVID-19 deaths: 10.0% of census tracts (*n* = 128) had no COVID-19 deaths and half of the census tracts (*n* = 652) had fewer than five deaths. The variance of the COVID-19 death rates (79.75) was substantially greater than its mean (6.61).

Table 1 summarizes the distribution of the COVID-19 Community Vulnerability Index (CCVI) for census tracts of Cook County. Compared with the U.S. census tracts median (0.50 for all themes), the theme median of the census tracts of Cook County ranked higher on Theme 7 Population density (0.89), Theme 2 Minority status & language (0.71), Theme 5 Health care system factors (0.66), and Theme 1 Socioeconomic status (0.64); Cook County census tracts ranked lower on the rest of the themes.

**Table 1:** Descriptive statistics for the COVID-19 Community Vulnerability Index (CCVI) for census tracts of Cook County (*N* = 1,284).

**Theme**	**Mean**	**Standard**	**Minimum**	**25^th^**	**Median**	**75^th^**	**Maximum**
		**deviation**		**percentile**		**percentile**	
1. Socioeconomic status	0.58	0.30	0.01	0.33	0.64	0.84	1.00
2. Minority status and language	0.68	0.21	0.03	0.52	0.71	0.85	1.00
3. Household and transportation	0.47	0.28	0.00	0.23	0.43	0.70	1.00
4. Epidemiological factors	0.42	0.23	0.03	0.23	0.41	0.61	0.98
5. Health care system factors	0.64	0.18	0.24	0.49	0.66	0.80	0.91
6. High risk environments	0.24	0.20	0.16	0.16	0.16	0.16	0.79
7. Population density	0.82	0.16	0.25	0.73	0.89	0.95	1.00

The correlations between the seven themes were weak to moderate. The Spearman's rank correlations among the CCVI themes ranged from −0.68 (between Themes 2 and 5) to 0.70 (between Themes 1 and 3). Table 2 summarizes the negative binomial regression results predicting the COVID-19 mortality rates at the census tract level with the seven CCVI themes. The two most significant themes were Themes 6 (High risk environments, incidence rate ratio (IRR) = 6.80), and Theme 4 (Epidemiological factors, IRR = 6.44). According to the model, the individuals living in census tracts with higher Theme 6 and Theme 4 were at a much greater risk of dying from COVID-19. Theme 2 (Minority status & language, IRR = 3.26) and Theme 5 (Health care system factors, IRR = 2.41) were also significantly associated with the COVID-19 mortality rate. The square of the Spearman Rank correlation between the actual deaths and predictions generated by the negative binomial regression was 52%. The predictive accuracy of a negative binomial model with the first three CCVI themes (non-COVID-19 specific themes) was only 32% (data not shown).

**Table 2:** Incident Rate Ratios (IRR) from the negative binomial regression model of the COVID-19 mortality rate (March 17, 2020 - March 16, 2021) (*N* = 1,284).

**Theme**	**Multivariate**	**Random forest**
	**IRR (95% CI)**	**Variable**
		**importance**
1. Socioeconomic status	0.96 (0.73, 1.25)	23.13
2. Minority status and language	3.26 (2.43, 4.37)	28.68
3. Household and transportation	1.61 (1.30, 2.01)	19.94
4. Epidemiological factors	6.44 (4.68, 8.87)	37.11
5. Health care system factors	2.41 (1.46, 3.97)	19.62
6. High risk environments	6.80 (5.66, 8.20)	44.73
7. Population density	0.93 (0.68, 1.25)	8.74

CI, confidence intervals; IRR, incidence rate ratio.

In addition, the Random Forest results (Table 2) confirmed the theme importance found in the regression model. The variable importance (Percent Increase in Mean Squared Error) of the three most important themes were: Theme 6 (44.73), Theme 4 (37.11), and Theme 2 (28.16). The predictive accuracy of the Random Forest method for COVID-19 deaths was 90%.

## Discussion

Our index validation approach allowed us to analyze the statistical significance and relative importance of seven themes explaining COVID-19 deaths during the first 12 months of the pandemic. Of the three most contributing themes, two were COVID-19 specific themes (6. High risk environments, 4. Epidemiological factors), and one was a general SVI theme (2. Minority status and language). This finding may suggest that the added disaster-specific indicators to SVI can improve the accuracy of the predictive model. We also observed that Theme 3 (Household and transportation) and Theme 5 (Health care system factors) were of importance. This finding confirmed other study findings revealing disparities in COVID-19 outcomes among communities with more racial/ethnic minorities in Cook County, who live in crowded housing, use public transportation for work, and have less healthcare access (1). Theme 1 (Socioeconomic status, IRR = 0.96) was not significantly associated with the COVID-19 mortality rate probably because it was moderately correlated with two other predictors, Themes 2 (*r*_*s*_ = 0.65) and 3 (*r*_*s*_ = 0.70).

The most significant predictor was Theme 6, High risk environments (the theme indicates the concentration of congregate settings, such as nursing homes, prisons, and crowded industries, which may lead to increased risk of transmission of COVID-19) (5). However, 86% of census tracts in Cook County had the same score for Theme 6. The three indicators of Theme 6 were measured at varying geographic levels: long-term care residents were measured at the census tract level, while prison populations and persons employed in high risk workplaces were measured at the county level (5). Because all Cook County census tracts have the same values for the latter two indicators (6), Theme 6 score variation was determined by the indicator of long-term care residents.

The significance of Theme 6 is consistent with study findings that long-term care residents have been one of the most vulnerable groups impacted by COVID-19 (1). However, it is uncertain how COVID-19 within long-term care facilities affects population-level COVID-19 outcomes in surrounding areas. Whether the high rates of COVID-19 deaths in long-term care facilities artificially inflated the COVID-19 mortality rates in otherwise less vulnerable areas requires further research.

The negative binomial regression and Random Forest showed good predictive ability in our analyses. Approximately 52% and 90% of the variability in mortality rates was explained by the seven CCVI themes. Random Forest outperformed other models due to its strength in fitting data with heterogeneity.

One of the limitations of our analysis is that we did not take into account the COVID-19 case rate. Because case rates were not available at the census tract level, estimating the case fatality was impossible. Second, our analysis was conducted at the census tract level to offer local-level knowledge for a better response to the pandemic. However, as the CCVI contains many county- and state-level indicators, a county-level analysis may reveal different trends.

Social vulnerability indices are an important tool for guiding emergency response and recovery efforts against disasters and infectious disease outbreaks. The COVID-19 pandemic has highlighted the critical need to identify the most vulnerable populations and provide support to mitigate risks. Beyond general measures of social vulnerability, disaster-specific indices could improve the predictive accuracy of disaster outcomes. However, given the difficulty in ascertaining many disaster-specific indicators at the local level, building a more robust local data infrastructure should be a priority. Hyper-local data can reveal local needs and vulnerabilities to inform more equitable disaster planning.

## Data availability statement

Publicly available datasets were used for this analysis. The data can be found: https://www.precisionforcoviddata.org/ and https://datacatalog.cookcountyil.gov/Public-Safety/Medical-Examiner-Case-Archive-COVID-19-Related-Dea/3trz-enys.

## Author contributions

JA led the analysis of the data and the writing of the manuscript. SH and SKo assisted in data analyses, interpretation, and manuscript development. SKi conceptualized and supervised the study and revised the manuscript. All authors contributed to the article and approved the submitted version.

## Funding

SKi was supported by the National Institute on Minority Health and Health Disparities (R01MD014839 and U54 MD012523).

## Conflict of interest

The authors declare that the research was conducted in the absence of any commercial or financial relationships that could be construed as a potential conflict of interest.

## Publisher's note

All claims expressed in this article are solely those of the authors and do not necessarily represent those of their affiliated organizations, or those of the publisher, the editors and the reviewers. Any product that may be evaluated in this article, or claim that may be made by its manufacturer, is not guaranteed or endorsed by the publisher.
