# Supplementary material for: Effectiveness of the COVID-19 Community Vulnerability Index in explaining COVID-19 deaths
Source: Front Public Health. 2022 Sep 23;10:953198. doi: 10.3389/fpubh.2022.953198 (PMC9539452; doi:10.3389/fpubh.2022.953198)
Supplement: Supplementary file 1 [file Table_1.docx]

**Supplemental Table.** CCVI Themes and Indicators

Surgo Ventures. COVID-19 Community Vulnerability Index (CCVI) Methodology 2020 [Available from: <https://precisionforcovid.org/ccvi>.]

| Themes | Indicators | Level |
| --- | --- | --- |
| 1. Socioeconomic status | Persons below poverty estimate | Census Tract |
|  | Civilian (age 16+) unemployed estimate | Census Tract |
|  | Per capita income estimate | Census Tract |
|  | Persons with no high school diploma (age 25+) estimate | Census Tract |
|  | Percent of population uninsured | Census Tract |
| 2. Minority status & language | Minority (all persons except white, non-Hispanic) estimate | Census Tract |
|  | Persons (age 5+) who speak English “less than well” estimate | Census Tract |
| 3. Household & transportation | Housing with structures with 10 or more units estimate | Census Tract |
|  | Households with more people than rooms estimate | Census Tract |
|  | Households with no vehicle available estimate | Census Tract |
|  | Persons in institutionalized group quarters estimate | Census Tract |
|  | Persons aged 17 and younger estimate | Census Tract |
|  | Single parent households with children under 18 estimate | Census Tract |
|  | Households without access to indoor plumbing | Census Tract |
|  | Mobile homes estimate | Census Tract |
|  | Civilian noninstitutionalized population with a disability estimate | Census Tract |
| 4. Epidemiological factors | Estimated percent of adults diagnosed with high cholesterol | Census Tract |
|  | Estimated percent of adults diagnosed with a stroke | Census Tract |
|  | Estimated percent of adults ever diagnosed with heart disease | Census Tract |
|  | Estimated percent of adults diagnosed with chronic obstructive pulmonary disease, emphysema, or chronic bronchitis | Census Tract |
|  | Estimated percent of adults reporting to smoke cigarettes | Census Tract |
|  | Annual cancer incidence per 100,000 people | County |
|  | Rate of persons living with an HIV diagnosis per 100,000 people | County |
|  | Estimated percent of adults reporting to be obese (a body mass index of 30 or greater) | Census Tract |
|  | Estimated percent of adults ever diagnosed with diabetes | Census Tract |
|  | Persons aged 65 and older estimate | Census Tract |
| 5. Health care system factors | Intensive Care Unit (ICU) Beds per 100,000 | County |
|  | Hospital beds per 100,000 | County |
|  | Epidemiologists per 100,000 | State |
|  | Agency for Healthcare Research and Quality-Prevention Quality Indicator Overall Composite: admission rates for preventable conditions adjusted per population | County |
|  | Health spending per capita | State |
|  | Aggregate cost of medical care | Census Tract |
|  | Percent of population with a Primary Care Physician | Census Tract |
|  | Total Public Health Emergency Preparedness (PHEP) Funding Per Capita | State |
|  | Health labs per 100,000 | County |
|  | Emergency services per 100,000 | State |
| 6. High risk environments | Long-term care (nursing homes, assisted living, and care homes) residents per 100,000 | Census Tract |
|  | Prisons population per 100,000 | County |
|  | Percentage of population employed in high-risk industry | County |
| 7. Population density | Estimated total number of people per unit area (sq. miles) | Census Tract |
